# Bird-biting mosquitoes on farms in southern England

**DOI:** 10.1136/vr.104830

**Published:** 2018-08-11

**Authors:** Victor Albert Brugman, Jolyon M Medlock, James G Logan, Anthony J Wilson, Steve W Lindsay, Anthony R Fooks, Peter P C Mertens, Nicholas Johnson, Simon T Carpenter

**Affiliations:** 1 Entomology group, The Pirbright Institute, Woking, UK; 2 Department of Disease Control, London School of Hygiene and Tropical Medicine, London, UK; 3 Department of Medical Entomology & Zoonoses Ecology, Emergency Response Department, Public Health England, Salisbury, UK; 4 Health Protection Research Unit in Emerging Infections & Zoonoses, Salisbury, UK; 5 Department of Biosciences, Durham University, Durham, UK; 6 Animal and Plant Health Agency, Weybridge, UK; 7 Department of Clinical Infection, Microbiology and Immunology, University of Liverpool, Liverpool, UK; 8 School of Veterinary Medicine and Science, The University of Nottingham, Sutton Bonington, UK; 9 Faculty of Health and Medical Sciences, University of Surrey, Guildford, UK

**Keywords:** entomology, mosquito, *Culex*, arthropodborne infections (arboviruses), biting rate

Mosquitoes that blood-feed on avian hosts are important vectors of many arthropod-borne viruses (arboviruses). In Europe, these include West Nile virus (WNV), Usutu virus (USUTV) and Sindbis virus.^1–3^ These are all maintained in enzootic bird-mosquito-bird cycles and are important veterinary and medical threats to the UK.^4 5^ Principally, veterinary concerns lie with the risks to domestic animals, such as the incidental spillover infection of horses with WNV which may lead to serious neurological sequelae.^6 7^ Wildlife may also be affected, with certain wild birds being highly susceptible to infection and death with USUTV,^8^ although poultry are less susceptible.^9 10^ To date, UK surveillance for these viruses has not yielded evidence of active virus transmission^11–14^ although serological evidence has been reported.^15 16^

Farms provide larval habitat for the development of a wide diversity of mosquitoes^17–19^ close to domestic animals and wildlife. Previously, we reported empirical data of mosquitoes on farms in the UK feeding on both resident and migratory birds.^20^ Additionally, some of these species feed on humans at farm sites,^21^ demonstrating the potential for spillover of viruses into these populations.

Studies using animal-baited traps provide data on the biting rates on key hosts.^22^ Several investigations using bird-baited traps (BBT) have been undertaken in Europe (eg, Czech Republic,^23^ France,^24 25^ Portugal^26^ and Sweden^27^) but UK data are limited to a single study.^28^ This investigation aimed to identify the ornithophilic activity of UK farm-associated mosquitoes using BBTs run alongside standard artificial surveillance traps.

The study was conducted between June and October 2013 on four mixed livestock farms in Oxfordshire, Kent, Hampshire and Surrey (see table 1 for habitat classifications according to Laird^29^). This region is considered to be at high risk of potential outbreaks as it is the warmest part of the country during the summer and early autumn when the biting activity of mosquitoes is likely to be highest. Trapping was conducted overnight (~12 hours) for nine nights on each farm using two BBTs, one set at 1 m and the second set at 4 m from the ground. A Mosquito Magnet Pro trap (MMP) (Midgetech, Stirling, UK) baited with 1-octen-3-ol was placed approximately 100 m away. A one-hour human landing catch (HLC) was additionally performed by one collector starting 30 minutes before sunset.

**Table 1:** Details of each farm together with habitat classifications present on each according to Laird,^29^ as follows: (1) flowing streams; (2) ponded streams; (3) lake edges; (4) swamps and marshes; (5) shallow permanent ponds; (6) shallow temporary pools; (7) intermittent ephemeral puddles; (8) natural containers; (9) artificial containers; (10) natural subterranean waters; (11) artificial subterranean waters

Farm location	Livestock present	General description	Habitat categories
Oxfordshire (51.714399, −1.389034)	Sheep, cattle, horses	Inland lowland farm surrounded by small villages and other agricultural holdings. Liable to spring and winter flooding due to proximity to the Thames.	1, 2, 5, 6, 7, 9
Kent (51.377201, 0.783809)	Sheep, cattle	Coastal grazing marsh in the Thames estuary. Large numbers of UK-resident and local migratory birds present.	1, 2, 4, 5, 6, 7, 9
Hampshire (50.822415, −0.952401)	Sheep, cattle	Coastal grazing marsh and mixed arable farm on Hayling Island.	1, 2, 4, 5, 6, 7, 9
Surrey (51.32052, −0.637904)	Cattle	Smallholding bordered by woodland and close to Her Majesty’s Prison Coldingley.	2, 6, 7, 9

**Figure 1:**
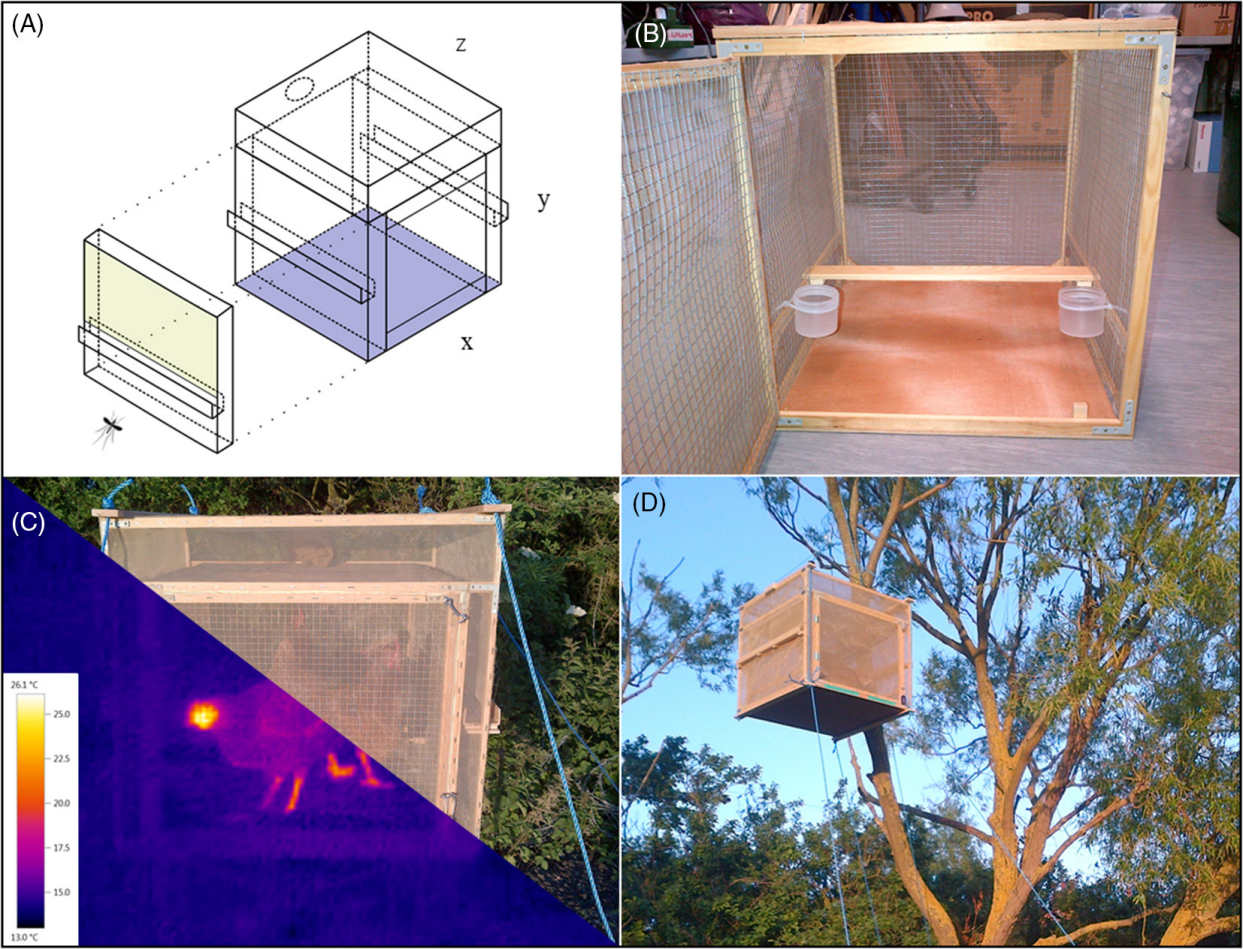
The bird-baited trap design: (A) isometric projection (produced using Google SketchUp) with one side additionally isolated to show the route of mosquito entry via the gutter baffle (x=700 mm, y=1200 mm, z=500 mm); (B) the interior portion of the trap showing double protective mesh, perch bar and food/water containers; (C) front view of trap with chickens inside, shown during the day and at night via thermal image taken with a Testo 875-1 Thermal Imaging Camera; (D) the trap secured using guide ropes in the high position (~4 m).

BBTs used chickens as bait and were constructed from pine stripwood, galvanised wire mesh and insect-proof netting (BioQuip, California, USA) (figure 1). Mosquitoes entered the trap via two gutter-like ‘baffles’ and were trapped in the top and side sections from where they were aspirated. The traps were modified from their original design^24^ following discussion with the Home Office where prevention of biting was recommended. Contact between chickens and mosquitoes was, therefore, prevented via an internal netting layer. Floor space was also increased, and a perch bar added to ensure the chickens were not stressed. Six chickens (ISA/Warren crossbreed) were maintained on a standard diet of layer pellets and two randomly allocated to each trap per evening; food (layer pellets and mixed corn) and water were provided throughout. Preliminary field testing, conducted inside an insect-proof tent (Insectopia, Austrey, UK) and using *Culex pipiens* sensu lato (sl) from The Pirbright Institute colony placed into the collection section of the trap overnight, showed that the BBTs retained 24–65 per cent of mosquitoes compared with 0–12 per cent when unbaited.

Collected mosquitoes were identified morphologically using standard keys.^30 31^ Specimens identified as *Cx pipiens* sl/*Cx torrentium* and *Anopheles maculipennis* sl were then delineated using previously described molecular methods.^20 32^

A total of 610 unfed female mosquitoes, of 16 species or species complexes, were collected (table 2). All farms, except the Oxfordshire site, yielded mosquitoes. The BBTs collected three species/species complexes: *Cx pipiens* sl/*Cx torrentium* (of all specimens collected in the study, 37/40 were *Cx pipiens* form (f) *pipiens*; three specimens were not fully identifiable), *Cx modestus* and *Coquillettidia richiardii*. The latter two species were also collected by HLC and in the MMP. Collectively, this supports their role as potential enzootic^20^ and bridge^21^ vectors for arboviruses in the UK,^5 33^ and validates the utility of the MMP as a tool for collecting them.^14^ The ornithophilic species *Cx pipiens* f *pipiens* was the most numerous species collected in the BBTs; it was, however, also collected by HLC at the Kent site, providing further evidence for the occurrence of human-biting by this ecoform in Kent.^21^

**Table 2:** The total number of mosquitoes collected during the study

Species	Kent farm				Hampshire farm				Surrey farm				Total
	BBH	BBL	HLC	MMP	BBH	BBL	HLC	MMP	BBH	BBL	HLC	MMP	
*Aedes geniculatus*	0	0	0	0	0	0	0	0	0	0	2	0	2
*Ae cantans/annulipes*	0	0	0	0	0	0	0	0	0	0	5	8	13
*Ae caspius/dorsalis*	0	0	1	7	0	0	1	1	0	0	0	0	10
*Ae detritus*	0	0	6	11	0	0	32	30	0	0	0	0	79
*Ae flavescens*	0	0	22	80	0	0	0	0	0	0	0	0	102
*Ae punctor*	0	0	0	0	0	0	0	0	0	0	35	9	44
*Ae rusticus*	0	0	5	2	0	0	0	0	0	0	0	3	10
*Aedes* species (damaged)	0	0	1	1	0	0	0	0	0	0	0	0	2
*Anopheles atroparvus*	0	0	4	0	0	0	0	0	0	0	0	0	4
*An claviger*	0	0	7	1	0	0	0	0	0	0	0	0	8
*An plumbeus*	0	0	0	0	0	0	0	0	0	0	1	1	2
*Coquillettidia richiardii*	0	2	23	141	0	0	0	0	0	0	0	0	166
*Culiseta annulata*	0	0	0	7	0	0	0	0	0	0	0	24	31
*Cu morsitans*	0	0	0	1	0	0	0	0	0	0	0	0	1
*Cu subochrea*	0	0	0	0	0	0	0	0	0	0	0	3	3
*Culex modestus*	0	1	42	50	0	0	0	0	0	0	0	0	93
*Cx pipiens* form (f) *pipiens*	8	6	3	14	2	0	0	2	0	1	0	1	37
*Cx pipiens* sensu lato (sl)*	0	0	0	0	0	1	0	0	0	0	0	0	1
*Cx pipiens* sl*/Cx torrentium*†	0	1	0	1	0	0	0	0	0	0	0	0	2
Totals per farm	8	10	114	316	2	1	33	33	0	1	43	49	610

No mosquitoes were collected from the Oxfordshire farm, therefore this site is omitted from the table.

*Specimens separated from Cx torrentium but could not be separated to ecoform.

†Specimens which could not be separated.

BBH, bird-baited trap ‘high’ position; BBL, bird-baited trap ‘low’ position; HLC, human landing catch; MMP, Mosquito Magnet Pro trap, baited with 1-octen-3-ol.

The MMP collected the greatest number of mosquitoes overall (n=398), while the BBTs collected far fewer specimens (n=22), averaging 1.00 mosquito/trap/night (range 0–6) in Kent, 0.17 (0–2) in Hampshire and 0.06 (0–1) in Surrey. These numbers were too low to permit a comparison between trap heights (high position n=10, low position n=12). Vertical stratification of mosquito populations has been reported across Europe, including the UK,^23 25 28 34 35^ although results are difficult to compare directly between the different trapping strategies used. Here, the absence of many mosquitoes in the BBTs, despite their collection by other methods, may reflect a low intrinsic ornithophily, the unattractiveness of chickens to these species (although chickens are widely used as arbovirus sentinels^16 36^), or most likely result from the constraints of trap design. *Anopheles atroparvus*, for example, was previously found to feed on chickens in Kent^20^ but here was absent from the BBTs. *Anopheles* species generally fly upwards upon hitting a vertical surface^37^ and thus the gutter design may have lessened the chances of entry for mosquitoes of this genus. Furthermore, unlike in the original design, mosquitoes were prevented from feeding on the birds which may have resulted in greater escape from the trap, as shown in other studies^38 39^ and as indicated by the variability in observed retention rates in the preliminary experiments. The recorded numbers may, therefore, be underestimates of true ornithophilic mosquito activity on these sites. Conversely, the numbers do fall within the range of the previous UK bird-baited trapping study which reported a combined mean of 1.05 mosquitoes/night for *Cx pipiens* sl and *Culiseta morsitans*.^28^

Despite the challenges of using animal-baited mosquito traps, the data generated using BBTs in this study are important to complement and validate data on mosquito host-seeking and feeding behaviour gained from surveillance studies, intensive HLCs^21^ and blood meal analyses.^20^ The results also demonstrate that farms with the same apparent habitat types present (Kent and Hampshire) may support a vastly different mosquito species diversity. Collectively, the ornithophilic and anthropophilic behaviour of farm-associated mosquitoes highlights their potential importance in enzootic and bridge arbovirus transmission in the event of a UK outbreak. Given current concerns regarding the invasion of exotic arboviruses,^40^ it would be prudent to increase awareness among the equine veterinary community in particular of clinical signs of mosquito-borne arboviruses in horses. These workers can play a key role in maintaining expertise in the wider community^41^ and offer preventive advice in the event of an outbreak. The simplest practical control measure targeted at mosquitoes would be to regularly empty stagnant water sources to disrupt larval habitats,^42^ which would be particularly important in reducing populations of key vector species *Cx pipiens* sl.^43 44^
